# X-ray Photoelectron Spectroscopy (XPS) Analysis of Nitrogen Environment in Small Extracellular Vesicle Membranes: A Potential Novel Technique with Application for Cancer Screening

**DOI:** 10.3390/cancers15092479

**Published:** 2023-04-26

**Authors:** María Sancho-Albero, Ana Martín-Pardillos, Silvia Irusta, Víctor Sebastián, Vicente Luis Cebolla, Roberto Pazo-Cid, Pilar Martín-Duque, Jesús Santamaría

**Affiliations:** 1 Instituto de Nanociencia y Materiales de Aragón (INMA), CSIC-University of Zaragoza, 50018 Zaragoza, Spain; 2 Department of Chemical Engineering and Environmental Technologies, University of Zaragoza, 50018 Zaragoza, Spain; 3 Networking Research Center on Bioengineering Biomaterials and Nanomedicine (CIBER-BBN), 28029 Madrid, Spain; 4 Instituto de Investigaciones Sanitarias de Aragón (IIS Aragón), 50009 Zaragoza, Spain; 5 Laboratorio de Miscroscopia Avanzadas, University of Zaragoza, 50018 Zaragoza, Spain; 6 Instituto de Carboquímica, ICB-CSIC, 50018 Zaragoza, Spain; 7 Medical Oncology Service, Miguel Servet Hospital, 50009 Zaragoza, Spain; 8 Instituto Aragonés de Ciencias de la Salud, 50009 Zaragoza, Spain; 9 Fundación Aragonesa para la Investigación y el Desarrollo (ARAID), 50018 Zaragoza, Spain

**Keywords:** XPS spectroscopy, extracellular vesicles, cancer, screening

## Abstract

**Simple Summary:**

In this study, X-ray Photoelectron Spectroscopy (XPS) is used as a fast technique to characterize the composition of nanosized small extracellular vesicles (EVs), with the aim of evaluating its possible application in cancer screening and diagnosis. This spectroscopic technique is an ideal approach to characterize membranes due to its sensitivity and its penetration depth, in the nanometric range, meaning that the results will mainly correspond to the membrane composition, with little interference from the biomolecules in the EV´s lumen. We propose the use of XPS to analyze the membranes of small extracellular vesicles isolated from bodily fluids of cancer patients and compared them to those of healthy cells and donors. Notably, we have focused our analysis on the N chemical environment of EV membranes, as this can be related to previous complex chemical analyses of lipid and protein contents in EVs. We provide preliminary results on a fast and non-invasive novel strategy, as a method or tool for research-based EV investigations with potential application in cancer screening and monitoring.

**Abstract:**

Small extracellular vesicle (EV) membranes display characteristic protein-lipidic composition features that are related to their cell of origin, providing valuable clues regarding their parental cell composition and real-time state. This could be especially interesting in the case of cancer cell-derived EVs, as their membranes could serve as valuable tools in liquid biopsy applications and to detect changes in the tumor malignancy. X-Ray Photoelectron Spectroscopy (XPS) is a powerful surface analysis technique able to detect every chemical element present, being also sensitive to their chemical environment. Here we explore the use of XPS as a fast technique to characterize EV membrane composition, with possible application in cancer research. Notably, we have focused on the nitrogen environment as an indicator of the relative abundance of pyridine-type bonding, primary, secondary and tertiary amines. Specifically, we have analyzed how tumoral and healthy cells have different nitrogen chemical environments that can indicate the presence or absence of malignancy. In addition, a collection of human serum samples from cancer patients and healthy donors was also analyzed. The differential XPS analysis of EVs collected from patients confirmed that the patterns of amine evolution could be related to markers of cancer disease, opening the possibility of their use as a non-invasive blood biomarker.

## 1. Introduction

Exosomes and extracellular vesicles (EVs) are membrane-based nanovesicles, that have been intensely investigated as excellent candidates for the diagnosis and the monitoring of several pathologies (such as cancer) due to their availability and established presence in most biological fluids [1]. These extracellular nanovesicles serve as delivery vehicles for the transport of a large range of active biomolecules between cells. They are composed of a cytosolic inner aqueous compartment surrounded by a protein-phospholipid bilayer membrane with similar biochemical and physicochemical characteristics to plasma membranes. However, in spite of the efforts invested in studying the physicochemical composition of EV membranes, the relationship between the produced EV membranes and the source parental cell is far from fully understood. 

It has been claimed that the membrane of cancer-derived EVs contains a fingerprint that on the one hand is responsible for their selectivity in targeting cells [2], and on the other hand, gives valuable clues for studying their parental cell composition. It seems reasonable to assume that cancer-cell derived EVs contain essential information regarding the cell of origin and the tumor environment in which it develops. In fact, EVs are currently being considered as potential biomarkers of some specific types of cancers [3]. For instance, exosomal miRNA has attracted attention for potential use as a diagnostic and prognostic marker in cancer. In this sense, Li et al. showed how miR-3591-3p could be used as a biomarker for glioma [4]. In another study, exosomal miR-423-3p was demonstrated to be a promising predictive biomarker for prostate cancer castration resistance development [5]. Other studies have used exosomal proteins as biomarkers in diagnosis. Chu et al. combined bioinformatic predictions with proteomic studies to conclude that higher expression levels of calsyntenin-1 (CLSTN1), clusterin (CLU) and neutrophil gelatinase-associated lipocalin (NGAL) proteins are solid serologic biomarkers for patients with lung adenocarcinoma [6]. Moreover, several studies have identified differential composition of exosomal lipids according to their malignancy [7,8,9,10,11,12]. For instance, Cheng et al. established that ovarian cancer-derived EVs contain higher levels of gangliosides, zymosterol, lysophosphatidylinositol, lysophosphatidylcholine, acca, lysophosphatidylserine, lysophosphatidylglycerol and cholesterol ester, and lower levels of ceramides, digalactosyldiacylglycerol, phosphatidylserine, phosphatidylinositol, phosphatidylglycerol, sphingomyelin, phosphatidylethanolamine, diacylglycerol and ceramides, compared with EVs derived from epithelial HOSEPiC cells [13]. Other studies confirmed that the lipid profile of pancreatic ductal adenocarcinoma (PDAC) was significantly dysregulated compared with healthy controls [14,15,16]. Our group has previously analyzed the lipidomic profile of healthy and tumoral metastatic and non-metastatic cell lines, revealing significant differences in the lipidomic profile of these cell lines according to their malignancy [7]. These data evidenced the potential role of exosomal lipids as diagnostic/prognostic markers in cancer. However, more clinical data are still required to confirm the diagnostic value of these techniques. Another drawback of analytical techniques based on genomics, lipidomics and proteomics is related to their time-consuming and expensive nature [11,13,17].

In view of the above, a strong interest exists in the development of alternative methodologies analyzing EVs as biomarkers of pathological processes. X-Ray Photoelectron Spectroscopy (XPS) is a high sensitivity surface analysis technique able to detect the relative abundance of each single element and also to give information regarding their chemical environment. A highly advantageous feature is the fact that the effective penetration of XPS for analysis is determined by the element being detected, the matrix or the orbital, and is limited to around 10 nanometers [18], meaning that (since the protein-lipidic membrane of EVs possesses a thickness about 5 nm) this technique will mainly “see” the membrane itself, and therefore the content of the EVs lumen (a lower concentration aqueous solution of biomolecules) will not interfere in the results. All of these reasons make XPS ideally suited to study EV membrane composition. 

We have explored the use of XPS as a fast research tool to characterize the composition of EVs, with the objective of assessing its possible application in cancer screening. We propose the use of XPS to analyze the membranes of EVs isolated from bodily fluids of target patients. Notably we have focused our analysis on the N chemical environment of EV membranes, as this can be related to previous complex chemical analyses of lipid contents in exosomes [7]. Specifically, we have analyzed EV membranes from cell cultures of different cancer cells (B16-F10 cells and B16-F1 cells) and compared them to EVs from healthy cells (NIH-3T3 fibroblasts and human placental mesenchymal stem cells (hpMSCs)) as controls. We have observed how tumoral and healthy cells have different nitrogen chemical environments that can be related to their malignancy. In order to assess the potential of this technique in cancer screening, a collection of human serum samples from patients and healthy donors was analyzed. The differential analysis of XPS patterns of EV membranes confirmed the significance of the nitrogen environment as a marker of ovarian and pancreatic tumors. Additionally, a clear correlation between CEA (a broadly used clinical cancer blood marker used currently in many diagnostic test) and the content of secondary amines in EV membranes of pancreatic cancer patients was detected. In summary, although XPS analysis cannot relate nitrogen environment with tumor progression, it gives a set of data that can open a new line of research regarding the different atomic environments of cancer EVs as a potential diagnosis/prognosis tool. These results open up interesting possibilities for the use of XPS as a new, fast and non-invasive cancer research technique useful for liquid biopsy and assessment of the nitrogen environment, describing a potential new biomarker. 

## 2. Materials and Methods

### 2.1. Cell Culture

Human placental mesenchymal stem cells (hpMSCs) were obtained from Cellular Engineering Technologies (CET) (Caralville, IA, USA), while B16-F1 (low metastatic variant) and B16-F10 (high metastatic variant) murine skin melanoma cells, were provided by cell services from Cancer Research-UK. NIH-3T3 murine healthy fibroblasts were obtained from Dr. Antonio de la Vieja’s group (Instituto de Salud Carlos lll). hpMSCs were cultured in Dulbecco’s modified Eagle’s medium (DMEM; Biowest, France) supplemented with 5 μg mL−1 of FGF-2 growth factor (PeproTech, Cranbury, NJ, USA), with 10% of fetal bovine serum (FBS, GIBCO, Waltham, MA, USA), 1% penicillin/streptomycin and 1% amphotericin (Biowest, France) and maintained at 37 °C in a 5% CO_2_-humidified atmosphere under hypoxic conditions (3% O_2_). For culturing B16-F1, B16-F10, and NIH-3T3 cells, DMEM with 10% of FBS (GIBCO), supplemented with 1% penicillin/streptomycin and 1% amphotericin (Biowest, France) was used. Finally, monocytes were cultured in RMPI Medium 1640 (Biowest, France) supplemented with 10% FBS (GIBCO), 1% penicillin/streptomycin and 1% amphotericin (Biowest, France). B16-F1, B16-F10 and NIH-3T3 cells were maintained under normoxic conditions. 

To obtain culture media free of EVs, they were depleted from serum by ultracentrifugation at 100,000× *g* for 8 h at 4 °C. 

### 2.2. Human Samples

Human serum samples and data from patients included in this study were provided by the Biobank of the Aragon Health System, integrated in the Spanish National Biobanks Network (PT20/00112), and they were processed following standard operating procedures with the appropriate approval of the Ethics and Scientific Committees. The present study (PI18/198) was approved by the human research review committee of the Research Ethics Committee of the Community of Aragon (*Comité de Ética de la Investigación de la Comunidad de Aragón*, CEICA), and was conducted in compliance with the ethical standards formulated in the Helsinki Declaration of 1996 (revised in 2000), upon obtaining the informed consent from all patients. 

Human serum samples were obtained from healthy donors (*n* = 10) and from stage III-IV pancreatic cancer patients (*n* = 10) using the first sample at diagnosis and two sub-sequential samples taken during disease progression. Ovarian cancer patients’ blood samples (*n* = 16) were obtained at diagnosis, including from patients in different stages (I, II, III, IV). Ovarian EV samples were divided for analysis according to the different stages, considering the possible variations of EVs depending on the disease stage. Samples were collected in vacutainer sodium citrate tubes in Hospital Universitario Miguel Servet, Zaragoza (Spain). Samples were stored at −80 °C until use. Then, samples were defrosted and 500 µL of serum was employed for isolation of the EVs and further XPS characterization. Blood markers, CEA and CA 19:9, were quantified by the National Health System, following standard procedures. The pancreatic samples used here for analysis of atomic composition and nitrogen environment are samples extracted at diagnosis, meaning, samples extracted before starting any treatment. Additionally, we have studied the evolution of secondary amines (first sample at diagnosis and two sub-sequential samples during disease progression) in relation to blood markers, CEA and CA 19:9. In this case, our purpose was to analyze if the variation of secondary amines occurs with the same trend as the evolution of blood markers, which are also modified depending on treatment. In this case, every patient has been studied independently, because we cannot compare secondary amine evolution during time among patients due to the differences in treatments and disease progression of each patient.

### 2.3. Isolation of EVs

EVs from cell culture samples were isolated following a protocol previously published and based on successive ultracentrifugation cycles from cell culture supernatants of the cells [19]. Briefly, cells were cultured until confluence and their supernatants were centrifuged for 20 min at 2000× *g* and at 4 °C (to remove remaining debris). For the elimination of the microvesicles, another centrifugation step was carried out for 1 h at 10,000× *g* and at 4 °C. To obtain the exosome fraction, the samples were ultracentrifuged for 2 h at 100,000× *g* and at 4 °C. A further washing step of exosomes with PBS was carried out to discard the co-isolated and adsorbed proteins on exosomes (2 h, 100,000, 4 °C). The exosomes pellet was finally resuspended in PBS for further analysis [1]. Some of these exosomes were previously described and characterized [2,20].

Ultracentrifugation is currently the gold standard for exosome isolation; thus far, some in vitro separation methods, such as ultracentrifugation, polymer-based exosome separation kits and immune affinity-based isolation using antibodies against exosome surface proteins, have been used for tumor exosome isolation [21]. We have compared the isolation efficiency between ultracentrifugation and affinity-based resin for serum samples. Due to the low volume of serum, the isolation efficiency by ultracentrifugation was too low to allow us to perform XPS analysis; therefore, we selected instead an affinity-based resin method to isolate exosomes from serum, but as the fraction collected might not be purely exosomes, we named all of them EVs. In the case of exosomes isolated from cell culture supernatants there is not a volume limitation and ultracentrifugation was the selected isolation procedure. In the case of human samples, serum samples (*n* = 56) were divided into control samples (*n* = 10 samples/patients), ovarian tumor samples (*n* = 16 samples/patients) and pancreatic tumor samples (*n* = 10 patients, 30 samples, 3 samples per patient, extracted at different time-points of the disease). EVs from serum samples (*n* = 56) were extracted using Plasma/Serum Exosome Purification Mini Kit, 50 Preps (57400, Norgen Biotek, Thorold, Canada). Following manufacturer instructions, the Plasma/Serum Exosome Purification Kit provides a reliable and convenient method to purify and enrich pure intact EVs factions from different plasma/serum samples, free from any cell-free circulating protein-bound RNA. Purification was performed according to the producer protocol. In brief, to 500 µL serum aliquots from each donor were added, in the following order, 3.5 mL nuclease-free water, 100 µL f ExoC buffer and 200 µL of Slurry E (resin). The mixture was mixed by vortexing and let stand at room temperature for 5 min, to allow the EVs to adhere to the resin. After incubation, the mixtures were centrifugated to recover the resin, the supernatant was discarded and the resin resuspended in 200 µL ExoR buffer; incubation was performed at room temperature for 5 min to release EVs from resin. After incubation, the mixture was centrifugated for 2 min at 500 rpm and the supernatant (containing free EVs) was recovered and filtered by centrifugation through a Mini Filter Spin column (1 min at 6000 rpm) to discard possible remaining resin (retained on the filter). After centrifugation, the EVs were ready for downstream applications. As negative control for following analysis, we used the same protocol starting with 4 mL nuclease-free water and the supernatant from the last step was analysed in the same way as the EVs from serum.

### 2.4. Transmission Electronic Microscopy (TEM) Sample Preparation and Analysis

The EVs’ morphology and the thickness of the EV membrane were characterized by transmission electron microscopy (T20-FEI Tecnai thermoionic transmission electron microscope) operated at 200 kV with a LaB6 electron source fitted with a “SuperTwin^®^” objective lens allowing a point-to-point resolution of 2.4 A [22]. EVs were stained with 3% phosphotungstic acid (Sigma Aldrich, St. Louis, MO, USA) in order to reveal their membrane structural details, as was previously described by our group. 

### 2.5. XPS Sample Preparation and Analysis

EV pellets isolated from cell cultures were finally resuspended in PBS and deposited drop by drop on a circular cover glass slip on a hotplate set at 30 °C. 

EVs from serum human samples and healthy controls were recovered in 200 µL ExoR buffer final volume. The resuspension was dried drop by drop (5 µL) on a circular cover glass slip on a hotplate set at 30 °C. 

The XPS analysis of all samples was performed 24 h after sample desiccation with an Axis Supra (Kratos Tech., San Diego, CA, USA) apparatus to determine the atomic percentage and the relative abundance of the different nitrogen species analyzed (R3-N, R2-NH/N-C, -NH2 and pyridine type bounding). The spectra were excited by the monochromatized Al Kα source (1486.6 eV) run at 10 kV and 8 mA. For the individual peak regions, a pass energy of 20 eV was employed. Survey spectra were measured at 120 eV pass energy. The CasaXPS software was used to analyze the peaks. After the subtraction of Shirley background, a weighted sum of Lorentzian and Gaussian components curve was employed.

### 2.6. Statistical Analysis

The results are expressed as mean ± SD (standard deviation). Statistical analysis of the data and the significant differences among the means were evaluated by two-way analysis of variance (ANOVA) and unpaired Student’s *t*-test for binary comparisons. Analysis was performed using GraphPad Software. Statistically significant differences were indicated as follows: * *p* < 0.05; ** *p* < 0.01; *** *p* < 0.0001 and **** *p* < 0.00001. 

## 3. Results

### 3.1. EV Characterization

**Figure 1:**
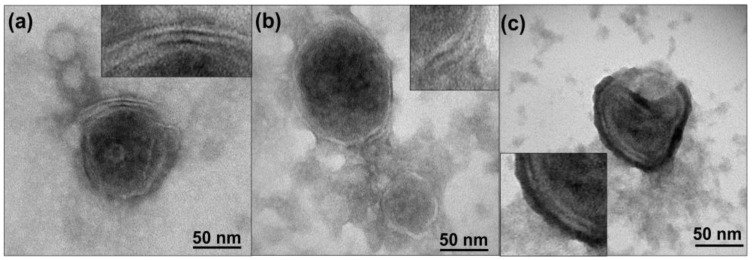
TEM images of isolated EVs from (**a**) B16-F10 cells, (**b**) hpMSCs and (**c**) NIH-3T3 cells. The characteristic EV bilipid membrane is observed in zoom insets.

EVs isolated from cell cultures exhibited a characteristic spherical shape with a diameter between 50 and 100 nm. TEM images depict the characteristic bilayer protein-lipidic membrane which was successfully revealed due to the negative staining process where H_3_PW_12_O_40_ particles are adsorbed in EVs surfaces (Figure 1). The EVs’ average thickness was around 4.5 to 5 nm, which is in agreement with the previous measurements from AFM analysis and described in other works Figure 1 includes images of EVs isolated from B16-F10, hpMSCs, and NIH-3T3 cells. 

### 3.2. XPS Analysis of Cell Culture EVs

**Figure 2:**
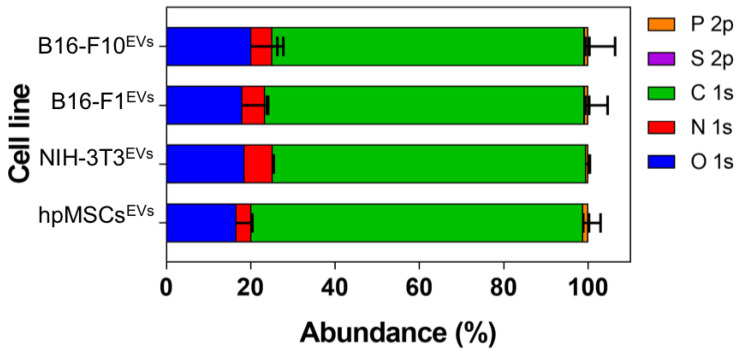
Atomic composition of EVs isolated from cell cultures.

First, the elemental composition of hpMSCs^EXOS^, NIH-3T3^EXOS^, B16-F1^EXOS^ and B16-F10^EXOS^ was evaluated by XPS. XPS analysis (Table S1 and Figure 2) shows the relative abundance of all the analyzed samples. As expected, carbon and oxygen were the most abundant elements present in the samples. As well, a significant amount of nitrogen was observed in all the EV samples. Finally, phosphorus was present in all the samples whereas only in some of them could a small amount of sulfur be found. 

The data revealed no significant differences in the atomic percentage of oxygen, nitrogen, carbon or phosphorus between EVs from healthy cells and cancer cells. There were no obvious differences between cells from the same background but with different grades of malignancy (B16-F1 vs. B16-F10) (Table S1 and Figure 2).

**Figure 3:**
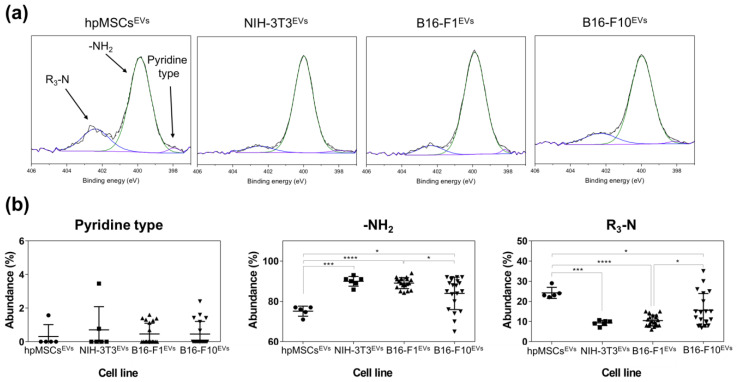
(**a**) XPS spectra of the nitrogen chemical environmental of EVs isolated from cell cultures. (**b**) Distribution of the nitrogen chemical environments according to the different cell lines. Statistically significant differences are indicated as follows: * *p* < 0.05; *** *p* < 0.0001 and **** *p* < 0.00001.

The above results seem to indicate that there are no intrinsic differences among EVs from healthy and cancer cell lines. In view of this, further analyses were focused on the N environment (Figure 3) since our previous results [7] using a rather complex approach (high-performance thin-layer chromatography-densitometry coupled to electrospray-tandem mass spectrometry) indicated that nitrogen-containing compounds could hold the key to the most promising features which could help distinguish EVs from various origins or degrees of malignancy. 

XPS analysis allows investigation of atomic environments in a fast and accurate way, since different N-containing functional groups present themselves with different energies in the spectrum. Table S2 and Figure 3b show the analysis of the nitrogen chemical environments of EVs from healthy cells (hpMSCs^EXOS^ and NIH-3T3^EXOS^) and from cancer cell lines (B16-F1^EXOS^ and B16-F10^EXOS^). Our results demonstrate that EVs from tumoral and healthy cells have different nitrogen chemical environments and that the N fingerprint can also be different among EVs from the same tumoral cell line depending on their malignancy degree. The results in Table S2 and Figure 3 indicate that secondary amines were absent in all EV samples. In addition, a trend towards a decrease in the proportion of primary amine groups (and a corresponding increase in the nitrogen associated to tertiary amines) can be observed as we move from EVs collected from fibroblasts (NIT-3T3) to low metastatic tumoral (B16-F1) and then to high metastatic tumoral cell lines. In particular, a clear trend for the diminution of the primary groups was observed when comparing the healthy and low metastatic tumoral exosomes. Indeed, statistically significant differences were obtained when comparing the low and the high metastatic tumoral EVs, evidencing how this biomarker can be potentially employed as a cancer monitoring tool. Interestingly, a high concentration of tertiary amine groups was also observed in EVs extracted from hpMSCs, in spite of their healthy nature. Of note, the highly metastatic B16-F10^EVs^ showed a nitrogen chemical environmental similar to stem cell derived EVs (hpMSCs^EVs^). This would be consistent with the known fact that aggressive cancer cells exhibit a phenotype similar to stem cells in terms of migration, proliferation and differentiation mechanisms. In this way, stem cells would be set apart from other healthy cells and this is also reflected in their nitrogen environment. Finally, pyridine-type nitrogen does not follow a clear trend and presents low but similar values for all cell types.

### 3.3. XPS Analysis of Serum EVs Isolated from Serum of Cancer Patients

It is obvious that EVs coming from tumoral cells only represent a small fraction of the EVs found in the blood of cancer cell patients. Since the methods available will isolate EVs released by all kind of cells from the body, a mixture of EVs from both cancer and healthy cells can be expected, with only a small fraction of EVs originating from tumoral cells. The dilution of cancer cell EVs by EVs from other origins represents the main difficulty when trying to detect their differential characteristics.

However, it seems possible that in cancer patients the production of tumoral EVs may increase depending on the stage of the disease. For this reason, we studied not only the percentage of nitrogen in different chemical environments but also the percentage of oxygen, nitrogen, carbon, phosphorus and the phosphorus/nitrogen ratio in EVs isolated from the serum of healthy donors and patients suffering from different grades of ovarian or pancreatic cancer. 

#### 3.3.1. XPS Analysis of Serum EVs Isolated from Ovarian Cancer Patients

The histopathological grade of ovarian epithelial carcinoma has generally been found to be of prognostic significance. The grading systems for epithelial ovarian carcinoma used most commonly have been those of the International Federation of Gynecology and Obstetrics (FIGO) and of the World Health Organization (WHO). The FIGO system is based on architectural features, and the grade depends on the ratio of glandular or papillary structures to solid tumor growth within an individual tumor; it has been related to molecular characteristics and clinical outcomes [23]. The patients included in this study were classified by tumor grade according to the FIGO system. EVs isolated from ovarian cancer patients were compared only with control female samples (*n* = 6), excluding male results, due to the possible influence of hormones in serum EV composition.

**Figure 4:**
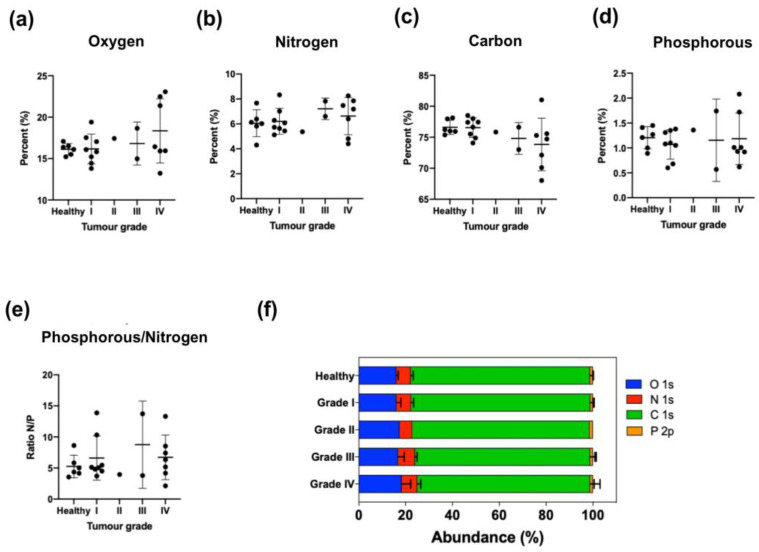
Atomic composition of EVs isolated from female control donors (*n* = 6) and ovarian tumor patients of different tumor grades: I (*n* = 8), II (*n* = 1), III (*n* = 2) and IV (*n* = 7). Mean ± SD. (**a**) Oxygen, (**b**) Nitrogen, (**c**) Carbon, (**d**) Phosphorus, (**e**) Phosphorus to nitrogen ratio, (**f**) Abundance (%).

As expected from our previous results (Figure 1, Table S1), carbon and oxygen were also the most abundant elements present in EVs isolated from serum, followed by nitrogen. Phosphorus was also found in all the samples but at levels slightly higher than 1%. The results presented in Table S3 and Figure 4f also confirmed that no significant differences existed in the percentage of oxygen, nitrogen, carbon, phosphorus or phosphorus/nitrogen relation as between EVs from healthy donors and from ovarian cancer patients even when the EVs were harvested at advanced stages of the illness (Figure 4a–e).

**Figure 5:**
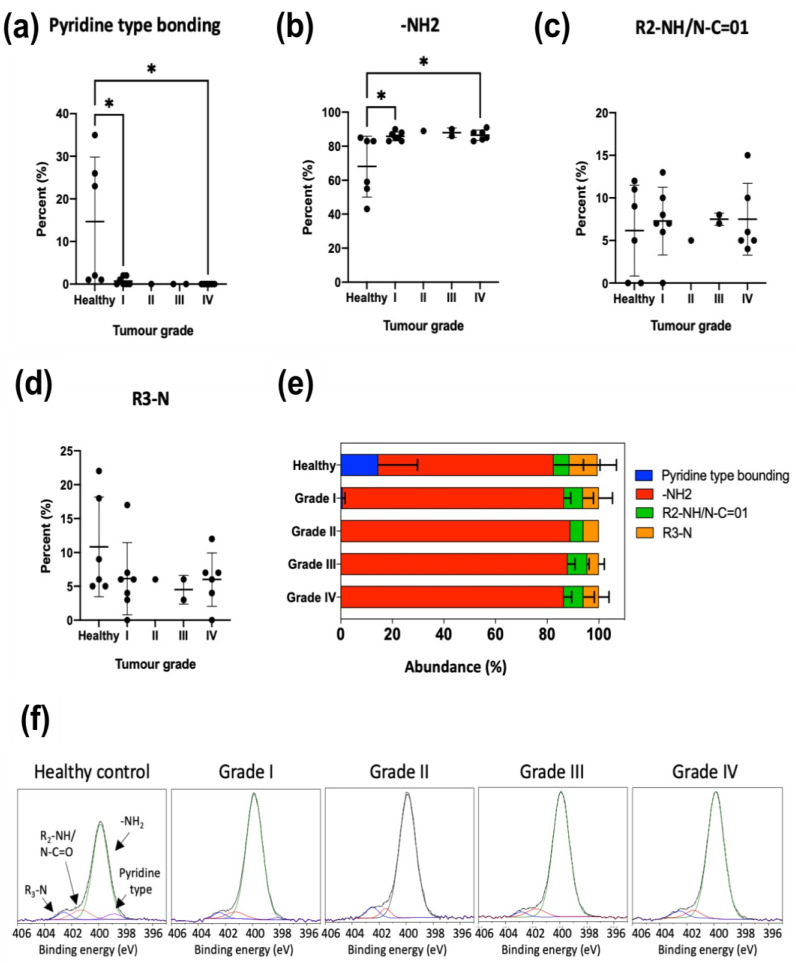
Superficial nitrogen composition of EVs isolated from female control donors (*n* = 6) and ovarian tumor patients of different tumor grades: I (*n* = 8), II (*n* = 1), III (*n* = 2) and IV (*n* = 7). Mean ± SD. (**a**) Pyridine-type bonding, (**b**) -NH_2_, (**c**) R_2_-NH/N-C=01, (**d**) R_3_-N, (**e**) Abundance (%), (**f**) XPS spectra of nitrogen chemical environment. Statistically significant differences are indicated as follows: * *p* < 0.05.

Encouraged by the in vitro results showing interesting differences in the nitrogen environment for tumoral and healthy cells (Table S2 and Figure 3), we carried out the same analysis for EVs isolated from serum of healthy and ovarian cancer patients. The results are presented in Table S4 and Figure 5. Again, the different species were classified according to the binding energy of the peaks detected in the XPS spectra of the nitrogen chemical environment (Figure 5f). Primary amines (-NH2) again were the most abundant nitrogen species in the EVs isolated from serum (Figure 5e) with a noticeable jump between healthy and ovarian cancer patients, while no significant differences were observed among patients from grade I to grade IV. However, the most significant finding was the large decrease of pyridine-type bonding from healthy to cancer patients (Table S4, Figure 5a,b). It is also noteworthy that secondary amines (that were virtually absent in the EVs isolated from four different cultures of healthy and tumoral lines) are now well represented in serum EVs, reaching relative abundance levels of 5–7% for both healthy and cancer patients. This could be an indication of the wide variety of cells shedding EVs into the bloodstream but it could also reflect the limitations of EV isolation methods when dealing with a complex environment such as serum, teeming with proteins of different composition. However, our purification method is able to purify intact EVs, free from contaminants, and we can then confirm that the obtained results are specific for EV membrane compounds.

#### 3.3.2. XPS Analysis of Serum EVs Isolated from Pancreatic Cancer Patients

Pancreatic tumors were classified according the American Joint Committee on Cancer (AJCC) classification [24], which is based on size and extension of the principal tumor (T), propagation to lymphatic nodules (N) and metastasis (M). We analyzed a total of 30 EV samples from the serum of pancreatic tumor patients although, in this case, only stage III-IV samples were available. However, unlike for the ovarian cancer patients, from every pancreatic cancer patient we were able to analyze three samples, one sample at diagnosis and two additional samples at two different time-points of the disease.

**Figure 6:**
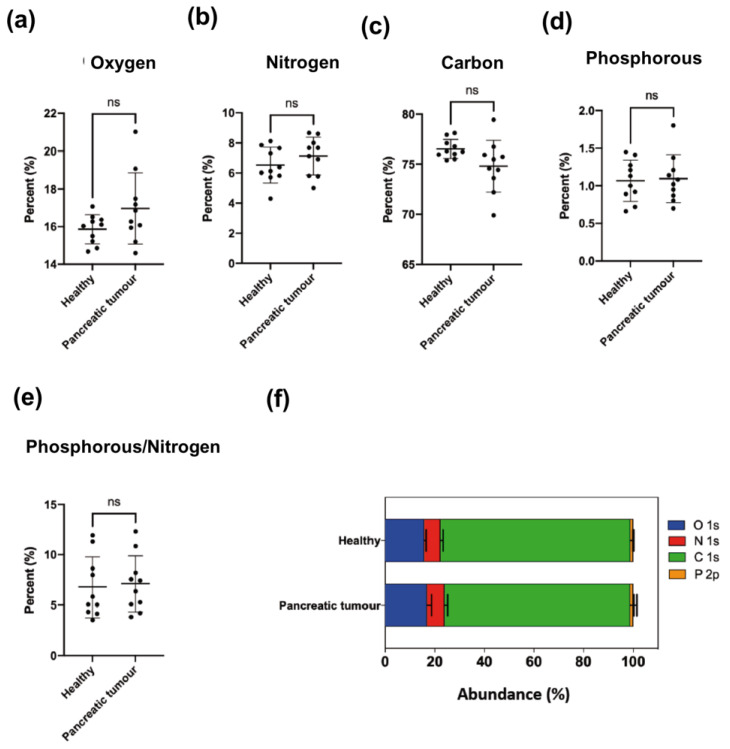
Atomic composition of EVs isolated from healthy donors (*n* = 10) and pancreatic tumor patients (*n* = 10) at diagnosis. Mean ± SD. (**a**) Oxygen, (**b**) Nitrogen, (**c**) Carbon, (**d**) Phosphorus, (**e**) Relation (ratio) between phosphorus and nitrogen, (**f**) Abundance (%).

To compare the content of different nitrogen compounds in EV membranes, we selected the first isolated sample of every patient, to avoid the influence due to treatments. Regarding elemental analysis, again carbon and oxygen were the most abundant elements followed by nitrogen and then phosphorus present in all the samples at around a 1% level. There were no significant differences in the relative abundance of these four elements between EVs isolated from healthy donors and pancreatic cancer patients (Table S5, Figure 6). 

**Figure 7:**
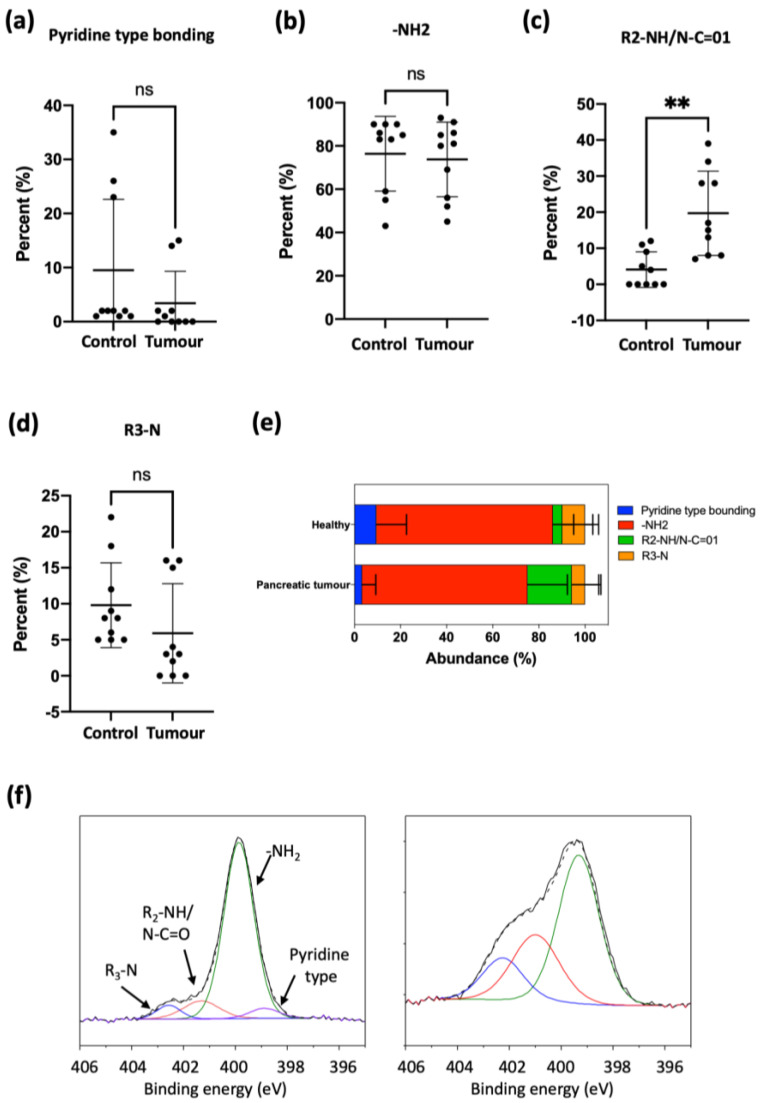
Nitrogen composition of EVs isolated from healthy donors (*n* = 10) and pancreatic tumor patients (*n* = 10) at diagnosis. Mean ± SD. (**a**) Pyridine-type bonding, (**b**) -NH_2_, (**c**) R2-NH/N-C=01, (**d**) R_3_-N, (**e**) Abundance (%), (**f**) XPS spectra of nitrogen chemical environment. Statistically significant differences are indicated as follows: ** *p* < 0.01.

Analysis of the nitrogen environment in serum EVs from pancreatic cancer patients in general gave a larger dispersion of data compared to ovarian cancer patients, and this makes it more difficult to detect significant differences, although some trends seem to be comparable. The main result of statistical significance concerns secondary amines (R*_2_*-NH/N-C=O) where a significant increase was observed compared to healthy donor samples (Figure 7b,c). This is in contrast with the results for ovarian cancer patients, where only a small, statistically non-significant increase was observed. Interestingly, although it was not statistically significant due to the high dispersion of data, we detected a tendency for a decrease in pyridine-type bonding in EVs isolated from pancreatic cancer patients compared to healthy donors (Figure 7a), similarly to EVs isolated from ovarian cancer patients (Table S4, Figure 5a,b). In addition, the concentration of tertiary (R*_3_*-N) amines was reduced on average by 40% in EVs isolated from pancreatic cancer patients compared to healthy donors (Figure 7c,d). The same trend was observed in ovarian cancer patients, although in both cases the high dispersion of data prevented statistical significance.

#### 3.3.3. Evolution of Secondary Amines and Clinical Tumor Blood Markers during Pancreatic Tumor Disease Progression

**Figure 8:**
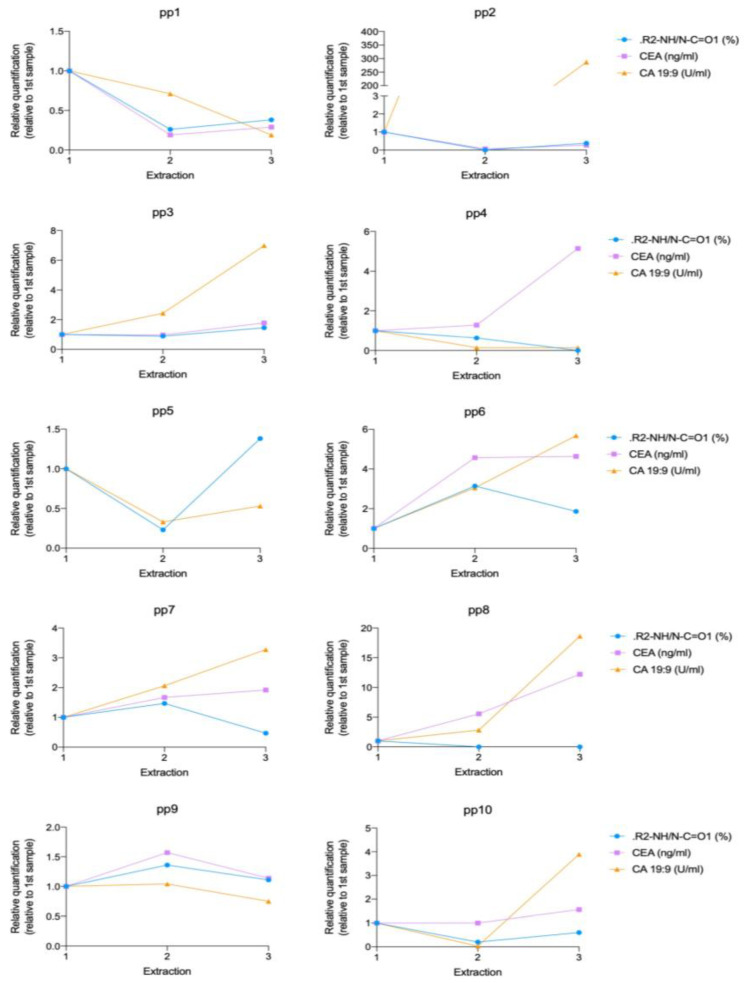
Evolution of secondary (R2-NH/N-C=01) and tumor blood markers, CEA and CA 19:9 during pancreatic tumor disease progression. Each graph corresponds to a specific patient (pp), showing the result of three sequential blood extractions. Absolute values were relativized to first time-point analysis value for every marker.

Since serum samples from the same patient at different times were available to us, we also analyzed the evolution of nitrogen-based markers (and specifically, of secondary amines that were the only parameter with enough statistical significance for pancreatic cancer patients) with time during the patient treatment time, and compared them to the evolution of clinical blood markers, CEA and CA 19:9 (Figure 8). Although CA19.9 is a well-established marker in the following up and prognosis of pancreatic cancer, CEA is not a fully validated marker, and is not universally accepted for this pathology. However, clinicians very often refer to this other marker for their prognoses. We decided to compare our XPS study with the value of those two markers in our patients. In this case, every patient has been studied independently, because it is not possible to compare secondary amine evolution over time among patients due to the differences in treatments and in disease progression of each patient. 

Comparative analysis of the secondary amines and both markers shows the same tendency for one or both markers and secondary amines in most patients. In summary, CEA was quantified in 9 out of 10 patients (Figure 8). In 6 out of these 9 patients (67%), the relative evolution of parameter values was analogous for secondary amines and CEA during the disease progression. 

CA 19:9 values were quantified in 10 patients, and 4 out of 10 patients (40%) showed the same relative trends for secondary amines and CA 19:9 during the disease progression. Although CEA and CA 19:9 are commonly used for the clinical diagnostic and prognostic analysis of pancreatic cancer, only 5 out of 9 (55%) cases showed the same tendency between the two of them. We can conclude that the correlation between CEA and secondary amines is higher than the correlation between the established clinical markers CEA and CA 19:9 (67% vs. 55%), at least for the cases studied here.

The correlation between secondary amines and CEA has been shown to be more robust than between secondary amines and CA 19:9 (67% vs. 40%). Therefore, it seems reasonable to conclude that secondary amines evolution during pancreatic cancer disease correlates well with one of the biomarkers (CEA) and could be further investigated as a new blood biomarker with a higher number of samples.

## 4. Discussion

The cell membrane is composed of lipids and proteins. The lipid fraction of the cell membrane consists mainly of phospholipids, glycolipids, sphingolipids and cholesterol [25]. The most abundant membrane lipids are the phospholipids. These have a polar head group and two hydrophobic hydrocarbon tails. The presence of carbon and oxygen is mainly related to the aliphatic chains in the tails. On the other hand, the main nitrogen contribution is associated with the polar groups of the phospholipidic head [26]. Cancer cells are notorious for the numerous adaptations that affect signaling cascades involved in therapy and immune system response; obviously, these involve changes in the type and/or amount of lipids that can be associated with the different cancer states. These changes are so prominent that lipid profile modifications could even be considered as a new cancer biomarker tool [25,27].

EVs are limited by a lipidic membrane, which encapsulates the cargo molecules in an inner aqueous core [28]. Lipid, and specifically phospholipids, are essential components of EV membranes and it is also known that specific lipids are enriched in EVs compared to their parent cells, playing indispensable roles on the structural and regulatory functions of EV biogenesis, release, targeting and cellular uptake [29,30,31]. Studies have showed that cholesterol, sphingomyelin, glycosphingolipids, phosphatidylserine, phosphatidylcholine are the most abundant lipids in exosomes [32]. It seems obvious that lipidomic studies are key to unravelling the biological relevance of EVs and will provide important clues on their functions [13].

We have recently proposed a robust analytical procedure to isolate EVs and obtain a wide-ranging and reliable analysis of the phospholipid (PL) content of their membranes [8]. Using this methodology, we have been able to analyze the lipidomic profile of healthy and tumoral metastatic and non-metastatic cell lines and patients by a novel methodology, revealing significant differences in the lipidomic profile of these cell lines [7]. Other recent studies using mass spectrometry platforms to analyze the lipidomic profile of EVs from urine have revealed significant differences between healthy control subjects and renal cell carcinoma patients [8], and for prostate cancer patients [9,10]. Similarly, differences in the lipidomic profile were detected among EVs isolated from plasma of healthy control, non-metastatic and metastatic colorectal cancer patients [11], hepatocellular carcinoma cancer patients [33] and lung cancer patients [12,34]. These results support the possible application of the EV lipidomic profile analysis as a diagnostic tool [32]. Additionally, the presence of EVs in urine or plasma provides significant advantages as a non-invasive tool for diagnosis [35]. 

These studies rely on the use of mass spectrometry protocols to analyze the lipidomic profile of EVs. While a powerful tool, these methods are rightly considered to constitute a complex technique, which requires the analysis of a complete lipidomic profile, distinguishing among the different lipids present in the EV membranes. The same could be said of our previous work involving EV isolation and analysis of the lipid EV extract by High-Performance Thin-Layer Chromatography-densitometry directly coupled to mass spectrometry, again a relatively complex procedure [7].

Instead, here we propose the characterization of the nitrogen environment using a single technique (XPS) and a relatively standard measurement. We have focused our analysis on nitrogen as this single element can be present in a variety of environments that can be easily analyzed by XPS, and their relative abundances can be used to characterize the evolution of the EV membrane composition. As already mentioned, XPS is ideal not only as a fast and reliable technique to identify different chemical environments of nitrogen atoms based on their binding energies, but also because being a surface technique that only responds to signals from the uppermost atomic layers, it has obvious advantages when one wants to analyze the membrane composition with minimal interference from the inner content of the EVs. 

The atomic composition of EV membranes isolated from cell cultures by ultracentrifugation (Table S1 and Figure 2) did not show significant differences among them. This is not surprising since the elemental composition analysis represents a high-level description of the sample and as such it is unlikely to detect differences in the chemical structure of the membranes (changing functional groups often gives rise to only small variations in elemental analysis). However, when the nitrogen chemical environments were considered, differences were detected between healthy and cancer cells (Table S2 and Figure 2 and Figure 3). Particularly, tertiary amines present in the EV membrane appeared to increase as the malignity of the parental source cells was higher. 

To further study this trend, EVs were isolated from human serum from healthy donors and from ovarian and pancreatic cancer patients (Tables S3 and S5, Figure 4 and Figure 6). Again, the elemental analysis did not show any significant differences, but analyzing the nitrogen environment allowed us to detect differences between healthy donor and ovarian and pancreas cancer patients (Tables S4 and S6, Figure 5 and Figure 7), confirming a modification of the lipidomic profile. However, in this case, the changes observed were different from those of the in vitro studies, the main difference being the disappearance of the pyridine-type nitrogen species.

Different studies have shown significant differences in the lipid profile of EVs from healthy and cancer-suffering persons [7,8,9,10,11,12]. However, a clear trend has not yet been found. Our knowledge about the lipid composition of cancer EVs is still scarce. Cheng et al. identified 30 lipid classes, including 1227 lipid species, in exosomes derived from ovarian cancer cells (SKOV-3) compared to those from ovarian surface epithelial cells (HOSEPiC). In particular, SKOV-3 derived EVs contained higher levels of many of the lipids studied than exosomes derived from HOSEPiC cells [13]. Our XPS results are effective in showing in a fast and simple measurement that EVs isolated from ovarian cancer patients show a strong decrease of pyridine-type bonding and also a noticeable increase of primary amines (-NH2) (Table S4, Figure 5), which implies that a significant alteration of the lipidomic profile has taken place, although identification of the specific lipid species altered would require a complex in-depth analysis.

Regarding PDAC, few studies relating diagnosis/prognosis and exosomal or EV lipid composition have been published. It was found that 1021 lipid species from different PDAC cell-derived exosomes (Panc-1, Capan-1, SW-1990, Mia PaCa-2, PPCL-68 and PPCL-46) and normal cell lines (hTERT-HPNE, HPDE-H6c7) were dysregulated between cancer-derived sEVs and normal sEVs, especially lipid species containing palmitic acid (16:0) and sphingomyelin [14]. In a genetic pancreatic mouse model, phosphatidylserine positive exosomes in blood were analyzed by ELISA, revealing a significant increase of these exosomes in PDAC-bearing mouse, suggesting the potential of phosphatidylserine positive exosomes for PDAC detection [15]. In another study by Tao et al., mass spectrometry was performed to analyze the lipid expression profile in exosomes derived from peripheral blood of PDAC patients and healthy patients. The authors found that about 270 lipids were significantly dysregulated between the exosomes of PDAC patients and healthy controls [16]. Again, these findings indicate the potential of lipids in exosomes from PDAC patients as biomarkers. Our own results for PDAC patient EVs show a significant increase of secondary amines (R_2_-NH/N-C=01) and also a trend towards the decrease of pyridine-type bonding, although without statistical significance (Table S6, Figure 7). As with ovarian cancer EVs, we do not identify specific lipids responsible for these changes, but XPS results provide a clear discriminating pattern, with a considerable simplification of the analysis for a potential liquid biopsy application. 

The fact that these general alterations of the nitrogen environment can be ascertained from EVs sampled from the blood of healthy and cancer patients is in itself remarkable, because cancer cell-originated EVs are necessarily diluted with EVs from every other kind of cell shedding EVs to the bloodstream and also because the EV isolation method based on columns is far from perfect and the EVs collected are likely to be contaminated with foreign nitrogen-containing species, such as plasma proteins. 

Finally, the analysis of the nitrogen environment was also useful to analyze pancreatic cancer evolution. This is an area of great interest and in fact there is not a universally accepted marker to characterize prognosis and response to treatment. We have compared the XPS results regarding the content of secondary amines with two blood markers, CA19:9 and CEA. Our results show that 67% of analyzed patients showed the same evolution of values for secondary amines and CEA and 40% showed the same evolution of values for secondary amines and CA 19:9 during the disease progression. The agreement found with CEA is better than the agreement between those established markers, since only 55% of patients showed a tendency for accordance between CEA and CA 19:9. We can therefore conclude from these preliminary results that the analysis of secondary amines’ evolution during pancreatic cancer disease has potential as a new PDAC biomarker and should be further investigated with a larger number of individuals.

## 5. Conclusions

In summary, analyzing by XPS the nitrogen environment of EVs isolated from the blood of two types of cancer patients reveals variations of pyridine-type bonding and primary amines in ovarian cancer, and secondary amines in pancreatic cancer, which can be related with significant changes in the lipidomic profile of their membranes, and provides analytical parameters that allow discrimination between cancer patients and healthy individuals. Furthermore, the analysis of secondary amines has shown potential as a marker of cancer evolution in pancreatic cancer. This type of measurement is attractive because it is relatively simple and could be used as a new, fast and non-invasive diagnostic tool. However, the differences found, while statistically significant in at least one nitrogen environmental parameter, do vary among the different types of cancer. It is known that exosome composition is dependent of cell origin [36,37]; for this reason, we could also expect variations in the exosome membrane. In consequence, the analysis of the nitrogen environment should be specific for every tumor type and, whenever possible, referenced to healthy donors of the same gender and age group. The data provided in this work support the consideration of XPS analysis as a new research tool for research-based EV investigators. Herein, we have identified the atomic composition of nitrogen species in EV membranes, opening a new line of research regarding the different atomic environments of cancer EVs as potential diagnosis/prognosis tools. 

Another important point to discuss is the possibility of contaminants coming from other cell types, including EVs from other strains. The tumor EVs analyzed here are collected from the blood together with EVs from immune and other cells. Indeed, we isolate the total pool of EVs from the sample, including those from cancer cells and they will be mixed with the rest. This is a comparative study where all the samples were processed equally and the presence of EVs from non-cancer cells will happen in all the samples, adding to the “background noise” and reducing the statistical significance of the results. Fortunately, significant differences between healthy and cancer patient samples were shown, meaning that in tumoral samples the pool would have been highly enriched with tumoral EVs, leading to sufficient differences to achieve statistical significance between samples. 

Further work is needed to validate the results with a wider set of clinical samples and also to identify, for each type of cancer, the relevant analytical parameters that could be used in an EV-based liquid biopsy scenario.

## Data Availability

The non confidential data can be shared up on request.

## Supplementary Materials

The following supporting information can be downloaded at: https://www.mdpi.com/article/10.3390/cancers15092479/s1, Table S1: Atomic percentage of EVs isolated from cell cultures. Mean ± SD; Table S2: Nitrogen chemical environmental analysis of EVs isolated from cell cultures. Mean ± SD; Table S3: Percentage atomic composition of EVs isolated from female control donors (*n* = 6) and ovarian tumor patients of different tumor grades: I (*n* = 8), II (*n* = 1), III (*n* = 2) and IV (*n* = 7). Mean ± SD; Table S4: Nitrogen composition of EVs isolated from female control donors (*n* = 6) and ovarian tumor patients of different tumor grades: I (*n* = 8), II (*n* = 1), III (*n* = 2) and IV (*n* = 7). Mean ± SD; Table S5: Atomic composition of EVs isolated from healthy donors (*n* = 10) and pancreatic tumor patients (*n* = 10) at diagnosis. Mean ± SD; Table S6: Nitrogen composition of EVs isolated from healthy donors (*n* = 10) and pancreatic tumor patients (*n* = 10) at diagnosis. Mean ± SD.
