# Supplementary material for: X-ray Photoelectron Spectroscopy (XPS) Analysis of Nitrogen Environment in Small Extracellular Vesicle Membranes: A Potential Novel Technique with Application for Cancer Screening
Source: Cancers (Basel). 2023 Apr 26;15(9):2479. doi: 10.3390/cancers15092479 (PMC10177571; doi:10.3390/cancers15092479)
Supplement: Supplementary file 1 [file cancers-15-02479-s001.zip › cancers-2235206-supplementary.pdf]

Table S1. Atomic percentage of EVs isolated from cell cultures. Mean±SD.

	Abundance (%)				
	**O 1s**	**N 1s**	**C 1s**	**S 2p**	**P 2p**
hpMSCs^{EVs}	16.56 ± 3.78	3.57 ± 0.22	78.64 ± 4.14	0.02 ± 0.03	1.19 ± 0.18
NIH-3T3^{EVs}	18.50 ± 0.78	6.70 ± 0.18	74.26 ± 0.88	0	0.53 ± 0.04
B16-F1^{EVs}	18.05 ± 5.77	5.37 ± 0.69	75.67 ± 5.51	0.10 ± 0.12	0.79 ± 0.25
B16-F10^{EVs}	20.18 ± 7.55	4.95 ± 1.18	73.99 ± 7.27	0.07 ± 0.12	0.80 ± 0.30

Table S2. Nitrogen chemical environmental analysis of EVs isolated from cell cultures. Mean±SD.

	Abundance (%)			
	**Pyridine type**	**-NH2**	**R2-NH**	**R3-N**
hpMSCs^{EVs}	0.31 ± 0.7	75.14 ± 2.50		24.22 ± 2.75
NIH-3T3^{EVs}	0.71 ± 1.38	89.98 ± 2,41		9.93 ± 1.31
B16-F1^{EVs}	0.46 ± 0.64	89.07 ± 2.76		10.47 ± 2.72
B16-F10^{EVs}	0.43 ± 0.73	83.97 ± 8.04		15.59 ± 8.29

Table S3. Percentage atomic composition of EVs isolated from female control donors (n=6) and ovarian-tumour patients of different tumour grades: I (n=8), II (n=1), III (n=2) and IV (n=7). Mean±SD.

	Abundance (%)			
	**O 1s**	**N 1s**	**C 1s**	**P 2p**
Healthy	16.13 ± 0.68	6.06 ± 1.08	76.61 ± 1.15	1.21 ± 0.22
Grade I	16.17 ± 1.80	6.21 ± 1.04	76.56 ± 1.58	1.07 ± 0.29
Grade II	17.43 ± 0	5.37 ± 0	75.84 ± 0	1.36 ± 0
Grade III	16.82 ± 2.60	7.21 ± 0.86	74.82 ± 2.57	1.16 ± 0.83
Grade IV	18.35 ± 3.88	6.63 ± 1.5	73.84 ± 4.25	1.18 ± 0.52

Table S4. Nitrogen composition of EVs isolated from female control donors (n=6) and ovarian-tumour patients of different tumour grades: I (n=8), II (n=1), III (n=2) and IV (n=7). Mean±SD.

	Abundance (%)			
	**Pyridine type bonding**	**-NH2**	**R2-NH**	**R3-N**
Healthy	14.50 ± 15.31	68.00 ± 17.97	6.17 ± 5.34	10.83 ± 7.36
Grade I	0.71 ± 0.95	85.86 ± 2.61	7.29 ± 3.99	6.14 ± 5.34
Grade II	0.00 ± 0.00	89.00 ± 0.00	5.00 ± 0.00	6.00 ± 0.00
Grade III	0.00 ± 0.00	88.00 ± 2.83	7.50 ± 0.71	4.50 ± 2.12
Grade IV	0.00 ± 0.00	86.50 ± 3.02	7.50 ± 4.23	6.00 ± 3.95

Table S5. Atomic composition of EVs isolated from healthy donors (n=10) and pancreatic tumour patients (n=10) at diagnosis. Mean±SD.

	**Abundance (%)**			
	**O 1s**	**N 1s**	**C 1s**	**P 2p**
Healthy	15.86 ± 0.77	6.54 ± 1.20	76.53 ± 0.95	1.07 ± 0.27
Pancreatic tumour	16.97 ± 1.89	7.13 ± 1.25	74.80 ± 2.59	1.09 ± 0.32

Table S6. Nitrogen composition of EVs isolated from healthy donors (n=10) and pancreatic tumour patients (n=10) at diagnosis. Mean±SD.

	**Abundance (%)**			
	**Pyridine type bounding**	**-NH2**	**R2-NH**	**R3-N**
Healthy	9.52 ± 13.11	76.55 ± 17.27	4.11 ± 4.93	9.82 ± 5.88
Pancreatic tumour	3.31 ± 5.91	71.79 ± 17.24	19.16 ± 11.66	5.74 ± 6.89
